# Computational Structural Biology of *S*-nitrosylation of Cancer Targets

**DOI:** 10.3389/fonc.2018.00272

**Published:** 2018-08-14

**Authors:** Emmanuelle Bignon, Maria Francesca Allega, Marta Lucchetta, Matteo Tiberti, Elena Papaleo

**Affiliations:** ^1^Computational Biology Laboratory Danish Cancer Society Research Center, Copenhagen, Denmark; ^2^Translational Disease Systems Biology, Faculty of Health and Medical Sciences, Novo Nordisk Foundation Center for Protein Research University of Copenhagen, Copenhagen, Denmark

**Keywords:** *S*-nitrosylation, (de)nitrosylating enzymes, redox modifications, molecular dynamics simulations, cysteine, redox cancer biology

## Abstract

Nitric oxide (NO) plays an essential role in redox signaling in normal and pathological cellular conditions. In particular, it is well known to react *in vivo* with cysteines by the so-called *S*-nitrosylation reaction. *S*-nitrosylation is a selective and reversible post-translational modification that exerts a myriad of different effects, such as the modulation of protein conformation, activity, stability, and biological interaction networks. We have appreciated, over the last years, the role of *S*-nitrosylation in normal and disease conditions. In this context, structural and computational studies can help to dissect the complex and multifaceted role of this redox post-translational modification. In this review article, we summarized the current state-of-the-art on the mechanism of *S*-nitrosylation, along with the structural and computational studies that have helped to unveil its effects and biological roles. We also discussed the need to move new steps forward especially in the direction of employing computational structural biology to address the molecular and atomistic details of *S*-nitrosylation. Indeed, this redox modification has been so far an underappreciated redox post-translational modification by the computational biochemistry community. In our review, we primarily focus on *S*-nitrosylated proteins that are attractive cancer targets due to the emerging relevance of this redox modification in a cancer setting.

## Introduction

**Figure 1:**
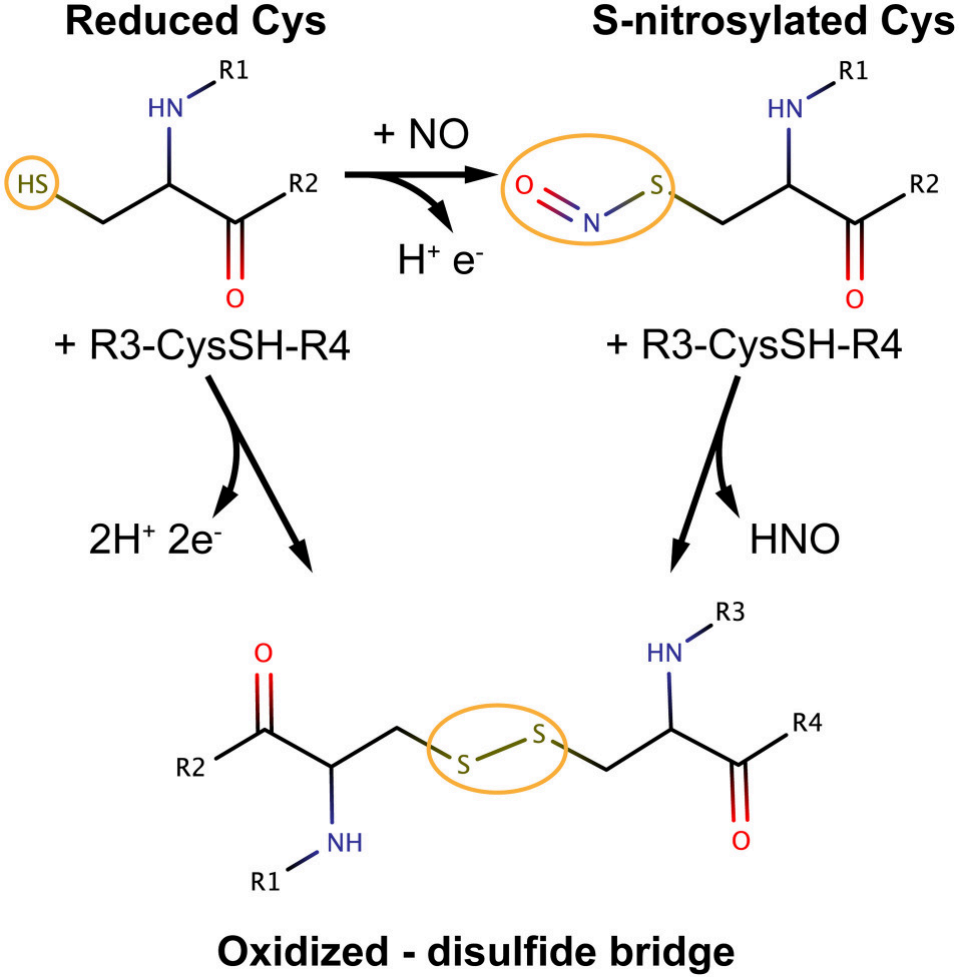
Most relevant cysteine oxidation states in the context of *S*-nitrosylation and possible pathways of interconversion. The figure is meant to be illustrative and does not depict the complete reaction schemes. R groups are part of the protein main chain.

Despite being amino acids (Cys) play diverse roles in biology. In fact, they represent a special class of residues due to the thiol moiety of their side chain (Figure 1). The thiol group can undergo a plethora of different biological modifications affecting protein structure, reactivity, stability and function (1). Cysteines are thus unique molecular switches (2). These modifications include, for example, disulfide bridge formation, high oxidation states, sulfenylation, persulfidation, metalation, *S*-nitrosylation, glutathionylation, sulfhydratation, among others. Cysteines can also be impacted by lipid modifications? including palmitoylation and prenylation or they can be the coordinating residues for metal ions such as zinc, iron, or copper in metallo-proteins.

Oxidative modifications of cysteine thiols can be a reversible or irreversible processes. Examples of reversible Cys modifications include Cys sulfenylation, *S*-glutathionylation and *S*-nitrosylation (SNO). With regards to the latter, NO is a reactive gas produced by NO synthases (NOS) 1–3 using the substrate arginine. NO produced inside the cell can be efficiently and quickly consumed through reactions with (bio)molecules that are in proximity to the NO source (3). A significant amount of the NO signal can be stored and propagated as nitrosyl adducts at specific cysteine sites of proteins via *S*-nitrosylation.

In this review, we will focus on computational structural and chemical studies that helped the understanding of the complex mechanisms induced by *S*-nitrosylation, as well as possible future directions for the computational studies of *S*-nitrosylation, which is a rather underappreciated modification compared to other well-known and more investigated post-translational modifications (PTMs), such as phosphorylation.

At first, we will provide the general background of what *S*-nitrosylation is, what is known on the enzymes regulating this PTM, as well as which are the biological effects so far discovered to be triggered by *S*-nitrosylation. We will then discuss the recent approaches and findings from bioinformatics, computational chemistry, and biochemistry methods applied to *S*-nitrosylation with particular attention to targets of interest for cancer research.

## *S*-nitrosylation of cysteine residues

Among the broad spectrum of cysteine redox modifications mentioned above, *S*-nitrosylation (SNO) accounts for the oxidative modification of cysteines by nitric oxide (NO) to form *S*-nitrosothiols (4). SNO is, by far, the reversible Cys modification with the most significant prevalence and cellular functions, providing a ubiquitous mechanism for cellular signaling mediated by thiols. NO exerts its primary biological functions through protein *S*-nitrosylation so that it can be considered the prototype of redox-based signals (4, 5).

*S*-nitrosylation is considered a reversible and ubiquitous PTM and many studies demonstrated its role in protein activity, stability, localization, and protein-protein interactions (see section Biological Mechanisms Promoted by *S*-nitrosylation) in a myriad of cellular processes (6). *S*-nitrosylation emerged in the last decades as a new paradigm in signal transduction and regulation of proteins (7). More than 3,000 proteins are known to be affected by this redox PTM (3).

Alterations of *S*-nitrosylated protein targets or the enzymes regulating SNO have been also associated with different pathologies, including cancer, cardiovascular, respiratory, and neurodegenerative disorders (4, 6, 8, 9).

Protein *S*-nitrosylation features tight spatiotemporal specificity for certain protein Cys residue (10, 11). If physiological amounts of NO are present, only one or few Cys residues of a protein are targeted. These modifications are generally sufficient to change the protein function, activity or specificity for interaction partners (11–13). *S*-nitrosylation, as well as other alternative *S*-oxidative modifications mediated by reactive oxygen species, can target separated populations of Cys residues. Thus, depending on the Cys that is modified, the functional effects that are triggered can be very diverse (14). *S*-nitrosylation has also been shown to target distinct Cys residues with the final goal of exerting a coordinated effect (4). The known and possible determinants of specificity of *S*-nitrosylation toward certain Cys residues are discussed more in details in section Computational Structural and Chemical Studies of *S*-nitrosylation. Computational studies allowed to disclose them and define sequence or structural motifs around SNO sites.

One major mechanism promoted by *S*-nitrosylation of proteins is based on *S*-transnitrosylation, i.e., the capability of modifying with SNO other protein targets thus allowing the propagation of the SNO-based signals (4), similarly to the well-known kinase-based signaling cascade.

Different degrees of *S*-nitrosylation can be observed in protein targets, spanning from cases of mono-SNO (single cysteine) to multiple cysteines (multi-SNO), depending on the availability of NO, as well as on the properties of the target proteins in the proximity of the Cys sites (3). NO production is tightly controlled in normal conditions, and this leads to a basal *S*-nitrosylation level. In this context, a subset of SNO targets (such as CD40 and pro-caspases) is in their resting state. Another subset of proteins, on the contrary, is constitutively *S*-nitrosylated. Indeed, they require this modification to be active, as exemplified by caveolin-1 or connexin-43. In cases in which these targets have multiple SNO sites, an incremental degree of *S*-nitrosylation can be observed with transitions from mono- to poly-SNO which allow a progressive activation in response to stress (3). The degree of SNO and the switch from mono- to multi-SNO can also strongly depend on the availability and accessibility of cysteines in the target protein. Cysteines are, for example, enriched in proteins that are on the cell surface or actively involved in cell-to-cell communication and signal transduction, such as CD40, other TNFRs, receptor tyrosine kinases, integrins, and connexins (3).

Due to the high SNO reactivity and the propensity for *S*-transnitrosylation, to fully appreciate the mechanisms and consequences of *S*-nitrosylation in normal and cancer cellular contexts is particularly challenging. Hence, it becomes crucial to assess the role of SNO-proteins as both targets of this redox modification and transducers of the SNO signal.

## Enzymatic regulation of *S*-nitrosylation

Cellular *S*-nitrosylation is dynamically governed by the equilibrium between *S*-nitrosylated proteins and low-molecular-weight *S*-nitrosothiols, which in turns are tightly controlled by several enzymes, such as S-nitrosylases and denitrosylases (4). NO is generally the product of three main isoforms of NO synthase (NOS) in mammalian cells (see section Nitric Oxide Synthases). *S*-nitrosothiols (RSNOs) are initially formed via different chemical routes that involve a one-electron oxidation, such as reaction of NO with thiyl radicals, transfer of the NO group from metal-NO complexes to a Cys thiolate, or the reaction of a Cys thiolate with species generated by NO auto-oxidation (4). Recent evidence suggested, however, an important role for metalloproteins in catalyzing *de novo S*-nitrosylation [see ref. (4) for more details]. The NO group is then transferred from a donor to an acceptor Cys thiol via *S*-transnitrosylation, (see section *S*-nitrosylation of Cysteine Residues). *S*-nitrosylation occurs not only in proteins but also in low molecular weight (LMW) thiols such as glutathione (GSH) and coenzyme A. SNO-proteins and SNO-LMW thiols exist in thermodynamic equilibria, which are regulated by the removal of the SNO moiety by the direct action of denitrosylases on the protein targets, as well as by the action of GSNOR on SNO-LMW molecules (see sections Denitrosylation Systems, Thioredoxin System and GSNOR System). These enzymes, described in the next sections, are crucial to control steady-state levels of SNO and to ensure the regulation of the NO-based signal cascade.

### Nitric oxide synthases

The compartmentalization of SNO targets with NOS enzymes favors the interaction between the enzymes and the *S*-nitrosylation substrate which can, in turn, occur directly, or through scaffolding proteins (4). For example, the CAPON protein acts as a scaffold to mediate the interaction between nNOS and the *S*-nitrosylation target Dexras1 (15). Another example is the complex formed by iNOS and S100A8/A9, for which targeted *S*-transnitrosylation is favored by S100A9 on multiple protein targets that share a short linear motif I/L-X-C-X2-D/E where C represents the SNO site (4).

Each NOS isoform can also be *S*-nitrosylated, generally through an auto-catalytic mechanism, which involves a metal center. NOSs can then act as *S*-transnitrosylating partners for scaffolding proteins with or without the intervention of a LMW-SNO (4).

The different NOS isoforms are expressed in various organs, tissues, cells, or even subcellular compartments (16). For example, the localization of eNOS in the Golgi apparatus allows the generation of a local NO pool that specifically targets the compartmentalized proteins for *S*-nitrosylation (17). An example of another finely tuned compartmentalization is attested by the binding of different NOS variants to separate regions of the same target protein, which result in *S*-nitrosylation of different Cys residues (4).

### Denitrosylation systems

**Figure 2:**
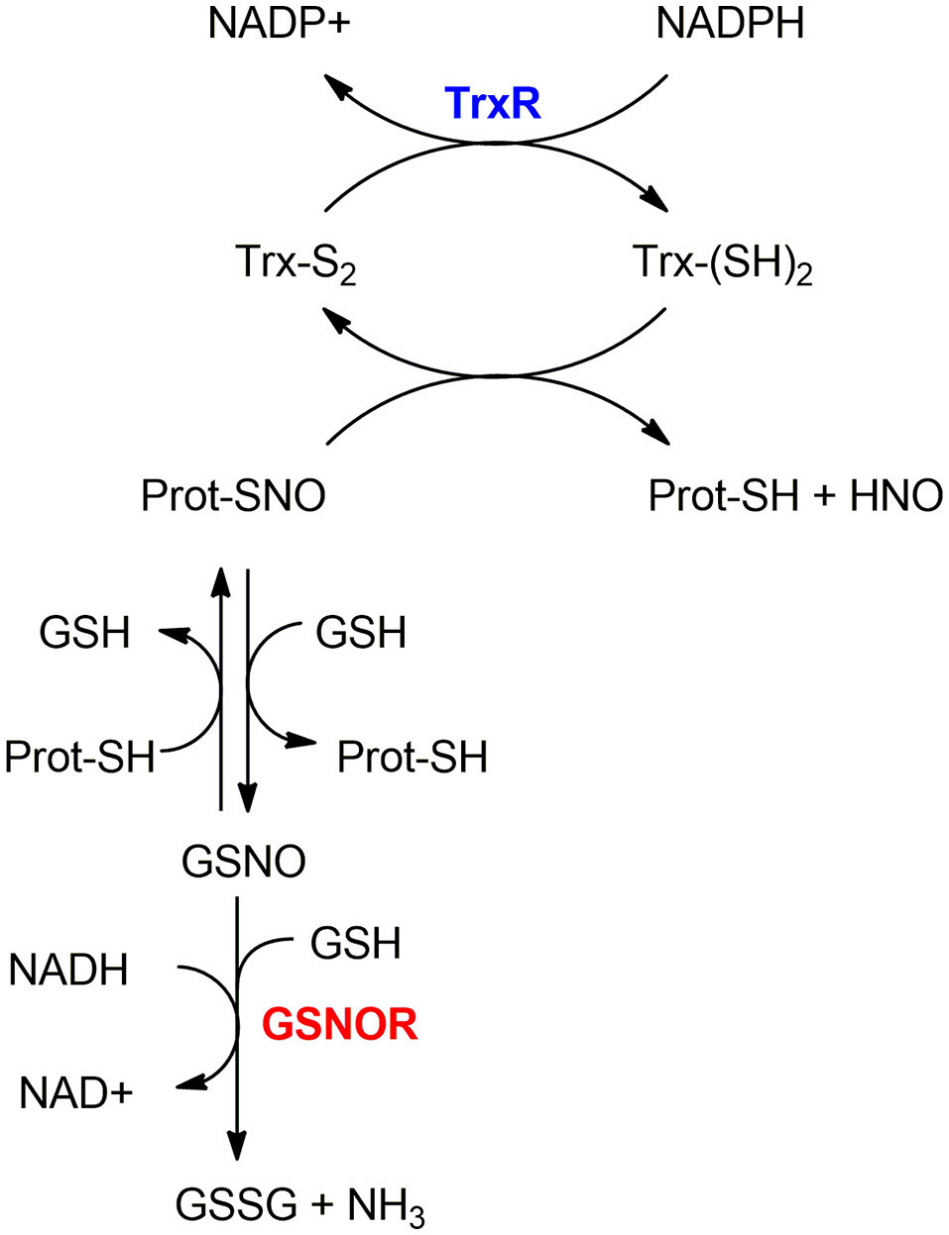
Mechanisms of denitrosylation. Thioredoxin-system (top): The reduced Thioredoxin (Trx(*SH*)_2_) denitrosylates the *S*-nitrosylated protein (Prot-SNO), releasing Prot-SH and the oxidized Trx (Trx(*S*_2_)), which is further reduced back to (Trx(*SH*)_2_) by Thioredoxin Reductase TrxR in a NADPH-dependent reaction. GSNOR-system (bottom): Prot-SNO is denitrosylated by *S*-transnitrosylation with glutathione (GSH), forming a reduced protein thiol (Prot-SH) and GSNO. The latter is then reduced by GSNOR to GSSG at the expenses of NADH and one GSH molecule. The resulting GSSG is further reduced by the Glutaredoxin enzyme (Grx) to give back the reduced GSH molecules—this step is not depicted here.

As mentioned in section S-nitrosylation of Cysteine Residues, *S*-nitrosylation is a PTM consisting of a covalent bond formation between of nitric oxide (NO) to a cysteine residue to form a *S*-nitrosothiol (5). In response to a specific stimulus, *S*-nitrosylated proteins (SNO-proteins) can undergo the reverse reaction of denitrosylation (i.e., reducing SNO back to SH). Two mechanisms of denitrosylation have been identified (18): the S-nitrosoglutathione reductase (GSNOR) system, comprising GSH and GSNOR, and the thioredoxin (Trx) system, comprising Trx and Trx reductase (TrxR) as shown in Figure 2. Both mechanisms use intermediate molecules (GSH and Trx) to remove the NO group from *S*-nitrosylated proteins. In the next paragraphs, we will illustrate these two denitrosylating systems and the cellular processes that they affect.

### Thioredoxin system

Thioredoxins (Trx) are a family of small redox proteins involved in multiple cellular processes.

The Trx system was originally identified as a key player in the cellular redox homeostasis thanks to its role as a disulfide reductase (19). There are two distinct mammalian thioredoxins, Trx1- mainly localized in the cytosol but possibly translocated to the nucleus-and Trx2, mainly located in the mitochondria.

The thioredoxin system is composed of a thioredoxin (Trx), a thioredoxin reductase (TrxR), and NADPH. It includes the dithiol Cys-X-X-Cys active site that is essential for their oxidoreductase function (20). In the denitrosylation process, the reduced Trx [Trx(SH)_2_] denitrosylates *S*-nitrosylated proteins forming a reduced protein thiol (-SH) and producing nitroxyl (HNO) or free NO groups and oxidized Trx [Trx(S-S)]; the latter is then reduced by TrxR at the expense of NADPH (21). For example, Trx1 reduces the target protein disulfide bond with concomitant oxidation of its Cys32 and Cys35 residues. TrxR then reduces the oxidized Trx1 thanks to NADPH consumption to regenerate the reduced Trx1 form.

Apart from its canonical role as a disulfide reductase, the Trx system has an important role in denitrosylation of *S*-nitrosylated proteins (21, 22). The Trx-dependent denitrosylation requires a multi-step process that entails: (i) the formation of mixed disulfide bridges through the attack of the nucleophilic Cys (Cys32 in Trx1) on the sulfur atom of the SNO moiety, (ii) the release of HNO, (iii) the resolution of the mixed disulfide bridge through the action of the second reactive Cys (Cys35 in Trx1) so that the oxidized variant of Trx can be formed and (iv) the final reduction step by the action of TrxR (19). The knowledge of this mechanism was also exploited experimentally to entrap a protein target in its *S*-nitrosylated state mutating the resolving Cys of Trx (Cys35 in Trx1) (23, 24).

An alternative mechanism for Trx-mediated *S*-denitrosylation has also been proposed in which the initial step is postulated to be a *S*-transnitrosylation reaction from the target protein to the active site cysteine of Trx, with the subsequent release of HNO (24).

Another protein, i.e., the thioredoxin-interacting protein (TXNIP), regulates the Trx denitrosylating activity. In a feedback loop, NO controls this inhibition by repressing TXNIP activity, providing a dynamic regulation of Trx-mediated denitrosylation in response to NO levels (25).

Moreover, attesting the versatility of the Trx system, Trx1 has been identified as a *S*-transnitrosylase (26). Trx1, in the oxidized state, can be modified by *S*-nitrosylation on its Cys73, which in turn mediates the *S*-nitrosylation of caspase-3 and other protein targets (19). The SNO-Trx form can be then regulated by either the Trx system or a GSH-mediated denitrosylation (21). In agreement with the existence of an acid-base motif for *S*-nitrosylation (see section *S*-nitrosylation Sites Specificity), it has been shown that charged residues in the proximity of Cys73 are required for Trx transnitrosylase activity (27). The stability of SNO-Trx is regulated by both Trx- and GSH-mediated denitrosylation (21). Additional Trx1 *S*-transnitrosylation motifs have been proposed that involve proximal alanine residues (28).

A thioredoxin-related protein of 14 kDa called Trp14 was also recently identified to act as a denitrosylase for the other master regulator of *S*-nitrosylation, i.e., GSNOR, as well as certain SNO-proteins, including caspase-3 and cathepsin B (24). Trp14 activity tightly depends on both TrxR1 and NADPH even if further studies are needed to better clarify Trp14 role *in vivo*.

Studies on targets of Trx-mediated denitrosylation allowed to identify two motifs, C-X5-K and C-X6-K, within the SNO targets that are modulated by the Trx system (28). These motifs could be used to predict and identify new targets where the Trx system is the major regulator of the *S*-nitrosylated state.

### GSNOR system

GSNOR (i.e., the GSNO reductase) is present in all mammals and ubiquitously expressed across different tissues (29). It is known as a class III alcohol dehydrogenase and it is encoded by the ADH5 human gene. GSNOR is a homodimer composed by two identical subunits (chains A and B) containing two bound zinc ions each (30).

The GSNOR-mediated protein denitrosylation requires the tripeptide GSH to form a reduced protein thiol (Prot-SH) and GSNO (18). GSNO is reduced by GSNOR to GSSG in a NADH-dependent reaction (using NADH as an electron donor) (31) involving hydride transfer. GSSG is further reduced by the Glutaredoxin enzyme (Grx) to give GSH back.

GSNOR expression and activity can be regulated by different biomolecules in a very context-dependent manner. For example, VEGF or IL-13 can induce GSNOR mRNA expression in lungs. Sp1 can transcriptionally regulate GSNOR levels in hepatocytes. MicroRNAs (miRs) such as miR-342-3p can downregulate GSNOR expression (4). Moreover, as also mentioned above, GSNOR is post-translationally regulated by *S*-nitrosylation and this modification has been suggested to regulate allosterically the enzyme activity, as attested by an enhanced GSNOR activity upon *S*-nitrosylation in mouse models (4).

The *S*-nitrosylated sites of GSNOR *in vivo* have not been identified yet and further studies will be required to fully understand and appreciate the mechanisms of this redox modification of the major regulator of *S*-nitrosylation. Prediction methods such as the ones described in section Prediction and Annotation of *S*-nitrosylated Proteins and the usage of molecular dynamics (MD) simulations could help in the identification of GSNO SNO-sites and in unveiling the determinants of its allosteric regulation. For example, the integration of computational techniques inspired by network theory and all-atom simulations hold promise to study long-range effects induced by PTMs (32–35). These methods could thus be translated to the study of GSNOR *S*-nitrosylation as soon as accurate physical models for *S*-nitrosylation will be available and validated.

As mentioned before, GSNOR has been found in different kind of tissues, particularly in liver, brain and kidney. GSNOR deficiency might positively or negatively affect physiology. GSNOR is the only ADH enzyme in the brain, highlighting its importance in this organ. As a result of its ubiquitous expression in different tissues, GSNOR controls several molecular processes and is likely to be involved in disease conditions onset.

### GSNOR in cancer

Nitric oxide (NO) regulates protein functions, as well as the activity of many enzymes. *S*-nitrosylation is a key mechanism in the transmission of NO-based cellular signals in vital cellular processes such as DNA repair (36), apoptosis (37), cell proliferation, and cell cycle regulation. These are all processes related to cancer onset.

As a key enzyme of denitrosylation, GSNOR controls the intracellular levels of *S*-nitrosylated proteins and the reduction of its expression or stability has been shown to result in dysfunctional *S*-nitrosylation signaling and, eventually, in pathological states such as cancer (38). In particular, GSNOR deregulation has been observed to be involved in some pathways representing the hallmarks of cancer like DNA damage repair, energetic metabolism and cell death. Noteworthy, GSNOR tumor suppressor role has been recently proposed (38).

It was demonstrated that GSNOR-deficiency is sufficient to induce spontaneous formation of hepatocellular carcinoma (HCC) (39) through *S*-nitrosylation and proteasomal degradation of the key DNA repair protein O(6)-alkylguanine-DNA alkyltransferase (AGT). The AGT enzyme removes the alkyl group from guanine bases, repairing the highly mutagenic and cytotoxic O6-alkylguanines which can be generated by carcinogenic compounds such as the diethylnitrosamine (DEN). Alkylation affects the stability of AGT resulting in its irreversible inactivation through degradation via proteasome. A similar effect is induced by *S*-nitrosylation of AGT on its Cys145; *S*-nitrosylated AGT is still degraded via the proteasome, but in this case before the repair of O6-alkylguanines. By GSNO catabolism, GSNOR maintains low levels of *S*-nitrosylated AGT. Therefore, GSNOR deficiency inactivates AGT-dependent DNA repair and may critically contribute to hepatocarcinogenesis (38).

Besides DNA alteration, tumor cells reorganize their core metabolism to sustain their growth and proliferation and GSNOR involvement in metabolic pathways has been observed. In particular, GSNOR deficiency in hepatocytes is characterized by mitochondrial alteration and by increases in succinate dehydrogenase (SDH) levels (40). Succinate dehydrogenase - or respiratory complex II - participates in both the citric acid cycle and the electron transport chain and catalyzes the oxidation of succinate to fumarate, regulating the levels of these two metabolites that are included in the class of “oncometabolites.” More precisely, the mechanism through which GSNOR ablation modulates SDH involves the mitochondrial molecular chaperone TNF receptor-associated protein 1 (TRAP1) which, if *S*-nitrosylated at Cys501, undergoes proteasomal degradation and is not able to interact with SDH, loosing the ability of inhibiting it (41). Besides, it was demonstrated that GSNOR ablation makes HCC cells more sensitive to SDH-targeting drugs (40), suggesting a new potential therapeutic target.

Different subtypes of breast cancers are also linked to the GSNOR expression modulation. In particular, the HER2 breast cancer subtype—characterized by high human epidermal growth factor receptor 2 (HER2) expression—is associated with lower expression of GSNOR which leads to a poor prognosis (42). Cañas et al. demonstrated that the antiproliferative effect of trastuzumab, a monoclonal antibody used for HER2 breast cancer, is suppressed by inhibition of GSNOR. Indeed, GSNOR restores the activation of survival signaling pathways, representing a possible reason for drug resistance observed in many patients on whom this treatment was used (43). Due to the central role of GSNOR in *S*-nitrosylation regulation and cancer, more insight will be gained in the future by comprehensive analyses of genomics and proteomics profiling of samples from cancer patients using biostatistics and bioinformatics to unveil the complex interplay between GSNOR, its regulators and targets in a cancer context.

## Biological mechanisms promoted by *S*-nitrosylation

As mentioned above, *S*-nitrosylation exerts a plethora of functions inside the cell. Indeed, *S*-nitrosylation gained attention as a PTM, but it is still less understood at the molecular level compared to other well-known PTMs. In 1992, Stamler et al. proposed for the first time that the formation of biologically active *S*-nitrosothiols—more stable than NO itself—could represent an important mechanism through which NO is involved in the regulation of cellular activities (44).

**Figure 3:**
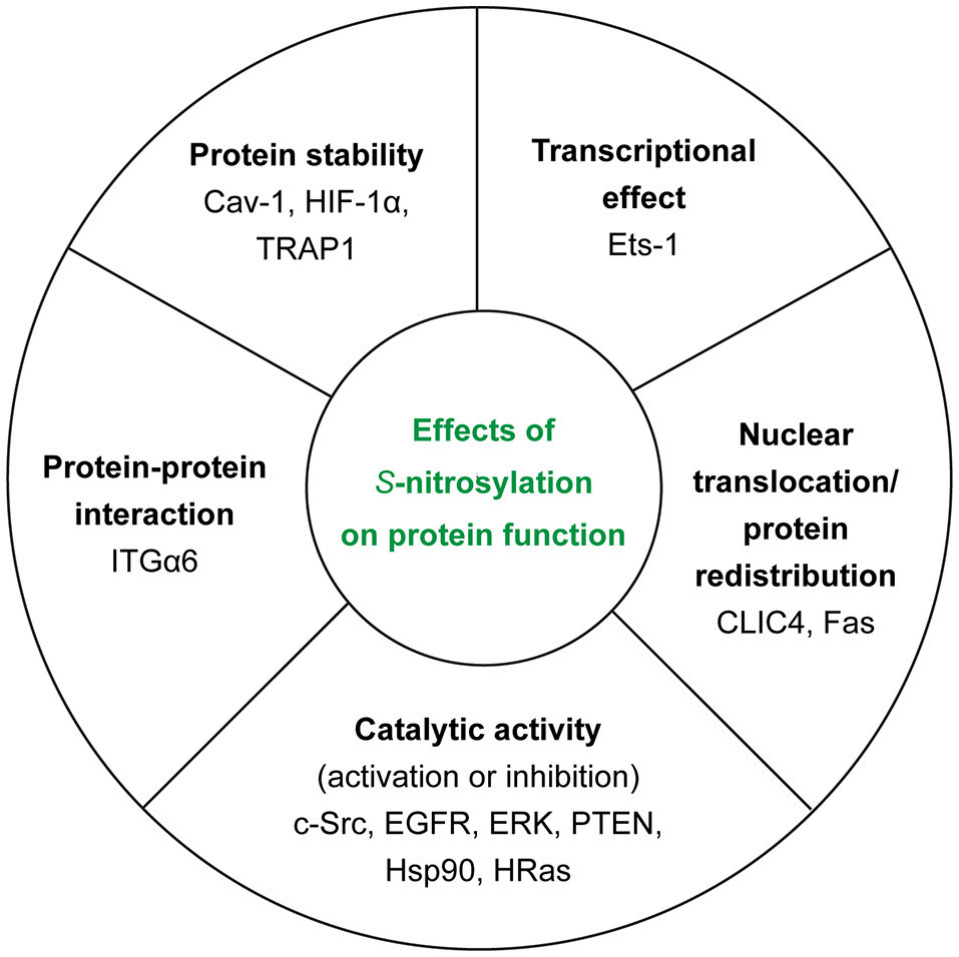
Effects of *S*-nitrosylation on protein function. For each class, we reported the examples of the target proteins that we discussed in the main text.

In the context of tumor biology, we know that nitric oxide (NO) has different effects on cellular ability to survive and proliferate depending on its concentration. In fact, high concentrations of NO (>500 nM) appear to be toxic for cancer cells—causing cytostasis and apoptosis—whereas low concentrations (< 100 nM) are able to induce the activation of cancer-promoting pathways (45). Thus, NO concentration—depending on the balance between NOSs activity and de-nitrosylation (see section GSNOR System)—determines also the extent of *S*-nitrosylation inside the cell. Another important source of NO is nitrite (46), especially in ischemic conditions but it is not the main focus of this section. In this section, we will try to summarize the polyhedral/multifaceted consequences of this PTM on protein function (Figure 3).

### Influence of *S*-nitrosylation on catalytic activity of enzymes

Probably one of the most common outcomes of S-nitrosylation is the regulation of catalytic activity of enzymes, whose modification can have activatory or inhibitory effects. In this way, the cellular redox state—highly variable/deregulated in cancer cells—exerts its role in controlling enzymatic activity.

*S*-nitrosylation can alter well-known cancer-related proteins, such as kinases. It has been observed in breast cancer cells that estrogens are able to work in synergy with NO to induce their proliferation and migration. In fact, in MCF7 cells, β-estradiol induces NOS expression and NO production, thus promoting the activation of c-Src through *S*-nitrosylation of Cys498 (47). c-Src is a tyrosine kinase, whose activation is responsible for disrupting E-cadherin junctions, promoting cell invasion and its catalytic activity is well-known to be regulated by phosphorylation. Recent evidence brought to light the existence of a tight regulation of c-Src based on *S*-nitrosylation and its cross-talk with phosphorylation (47).

The increase of iNOS (inducible NOS, also known as NOS2) is correlated with a decreased survival of ER negative and basal-like breast cancer (BC) patients. Indeed, this correlates with high EGFR phosphorylation levels. A study from 2012 showed that, in the context of basal-like BC, *S*-nitrosylation can stimulate EGFR and Src proteins (both membrane-associated proteins). The *S*-nitrosylated variants of EGFR and Src are then responsible for activating the oncogenic signaling based on c-Myc, Akt, STAT3, and β-catenin, while inhibiting the tumor suppressor PP2A (48). Moreover, NO levels required to activate these proteins are the same levels promoting an aggressive cellular phenotype (49).

More recently, it has been discovered that the NO-dependent EGFR activation can induce Extracellular signal-Regulated Kinase (ERK) phosphorylation—whose abnormal elevation has been described in tumor cells—thus activating it in basal-like triple negative breast cancer (50). ERK belongs to the MAPK superfamily and its aberrant upregulation and activation, which frequently occur in human tumors, are responsible for the acquisition of a malignant phenotype (51). In particular, there is a subset of basal-like BC, i.e., the BL2 molecular subtype, that is highly dependent on growth factor signaling (EGFR included) (52). In the BL2 subtype, Garrido et al. (50) demonstrated that NO-mediated activation of both EGFR and ERK was responsible for the increased migration and invasion abilities of cancer cells, this being accompanied by NF-kB activation and the increased secretion of pro-inflammatory cytokines.

ERK *S*-nitrosylation also links NO signaling to apoptosis. Indeed, ERK1 and ERK2 exert anti-apoptotic functions and are able, on the one hand, to induce the activity of other apoptosis antagonists, as for instance Bcl-2 and IAP. On the other hand, they can repress pro-apoptotic proteins, such as Bad and BIM (53). More in details, in a cellular model of NO-induced apoptosis, it has been demonstrated that NO decreases the levels of p-ERK, suggesting the *S*-nitrosylation of the kinase as an essential way to trigger cell death of tumor cells (54).

Recently, Gupta et al. (55) showed that *S*-nitrosylation plays a role also in the PI3K/Akt pathway, which is often dysregulated in cancer. In conditions of energy deprivation and in the presence of a signal able to activate the AMPK kinase, eNOS activation promotes inhibition of PTEN through its *S*-nitrosylation and degradation mediated by the ubiquitin-proteasome system. Intriguingly, PTEN inactivation upon *S*-nitrosylation was originally identified in the context of neurodegenerative diseases (56), whereas Gupta and coworkers, for the first time, demonstrated the role of this redox-dependent modification in supporting proliferation and survival of cancer cells through the activation of PI3K/Akt signaling and the subsequent stimulation of mTOR activity (55).

Heat Shock Protein 90 (Hsp90)—the cytosolic molecular chaperone—represents a co-activator of eNOS associating to the NO synthase together with Akt. Indeed, to exert this role, Hsp90 needs to work as an ATPase, but this activity was discovered to be inhibited by *S*-nitrosylation, occurring on Cys597, localized in the C-terminal domain in the region interacting with eNOS. In fact, it was proposed that the PTM induces a conformational change able to disrupt the interaction between eNOS and Hsp90. This may represent a mechanism to react to overproduction of NO inside the cells (57, 58). The *S*-nitrosylation of Cys597 also regulates ATP hydrolysis and chaperone activity of Hsp90 and shifts the conformational equilibrium within the ATPase cycle (58). Future studies in which the SNO modification of Cys597 can be properly modeled, with the related conformational changes assessed, will provide more details on the mechanism and interplay between this redox modification and the activation of the chaperone.

The class of Ras GTPases—HRas, NRas, and KRas, i.e., the founding members of a large superfamily of small GTPases—regulates several cytoplasmic signaling networks that govern cell growth, survival, and differentiation and act upstream to several pathways mentioned above (59). The three Ras proteins are over-activated by somatic mutations in 33% of human cancers, contributing to excessive growth, invasion, and ability to metastasize (60). The mutated variants of these proteins are associated with a constitutively active GTP-bound state, which makes cancer cells addicted to the expression of oncogenic Ras proteins. Some years ago, it has been also demonstrated that HRas undergoes *S*-nitrosylation on Cys118 with the effect of stabilizing the GTP-bound HRas form by enhancing the dissociation of guanine nucleotides (61). For this reason, it has been proposed that this PTM could represent a way to diversify the Ras-dependent oncogenic signaling beyond that of the mutated Ras.

In summary, many examples have been provided of regulation of enzymatic activity by *S*-nitrosylation, and they are especially important in the context of kinases, G-coupled proteins and chaperones i.e., three usual suspects in a cancer setting.

### Nuclear translocation/protein redistribution

A large body of literature investigates and explains the multi-faceted role of ion channels, both potassium and chloride, in the process of tumorigenesis. Indeed, they are involved in many of the main processes leading the transformation of a normal cell into a neoplastic one and, among all the ion channels, several chloride intracellular channels (CLICs) have been demonstrated to be overexpressed in different cancer types (62). Among the latter, CLIC4 has been originally identified as a p53- and cMyc-responsive protein with proapoptotic functions, most of which are dependent on its translocation to the nucleus. Moreover, this channel undergoes structural conformational changes upon cellular redox changes (63, 64). In 2010, Malik et al. (65) have demonstrated that *S*-nitrosylation affects CLIC4 nuclear translocation, enhancing the association of the channel protein with two proteins responsible for the nuclear import, i.e., importin α and Ran, and thus increasing the nuclear levels of CLIC4. It has been proposed that through this mechanism CLIC4 would be able to induce apoptosis when NO cellular levels overcome the denitrosylating capability of the cell. Moreover, this would explain the mislocalization of CLIC4 in tumor cells where the redox balance is altered (65, 66).

*S*-nitrosylation can be responsible not only for the protein nuclear translocation but also for protein redistribution inside the cell. This is the case of the Tumor necrosis factor receptor superfamily member 6 (also known as Fas/APO-1/CD95), a cell surface receptor able to induce apoptosis when stimulated by the ligand FasL/CD95L or agonistic antibodies. After stimulation, the receptor recruits a number of proapoptotic factors - including caspase-8/10 and procaspase 8/10 to name a few—to assemble the death-inducing signaling complex (DISC) (67). Cancer cells evolved several ways to elude the possible apoptotic induction by CD95, for instance regulating the expression of the receptor (68) or inhibiting the interactions between the members of the DISC complex (69). A few years ago, it has been demonstrated that Fas undergoes *S*-nitrosylation at Cys199 in the transmembrane domain and Cys304 in the death domain, with both the events involved in determining Fas membrane localization, the latter also favoring Fas aggregation and translocation in plasma membrane lipid rafts (70, 71). As already mentioned, cancer cells can lose sensitivity to Fas-mediated apoptosis because of a decreased Fas expression, but *S*-nitrosylation (and inducers of this PTM) may be able to recover this sensitivity (70).

### Transcription factors in NO-dependent signaling

As mentioned above, the Ras family of proteins—that owes its fame to the fact of being very often mutated in most of the cancer types—is a known target of *S*-nitrosylation (see section Influence of *S*-nitrosylation on Catalytic Activity of Enzymes). To fully appreciate the regulation of Ras family members and Ras-dependent tumorigenesis, another player needs to be considered, adding an extra layer of complexity to an already intricate scenario. In 2012 Ras was shown to activate Ets-1 transcriptional activity in human ER-negative (ER-) breast tumors (72). Ets-1 is a proto-oncoprotein member of the Ets family of transcription factors sharing a unique DNA binding domain. Being expressed in a large variety of cellular types, Ets-1 has a role both in physiological and pathological conditions but its role in carcinogenesis is due to its ability to regulate the expression of angiogenic and extracellular matrix remodeling factors promoting an invasive phenotype (73, 74). When performing the promoter region analysis of genes up-regulated in ER- breast tumors showing high levels of NOS2 expression, Switzer et al. observed that the common denominator of the promoters of these genes was the presence of the Ets-binding sequence, pointing at the role of this transcription factor in the NOS2 (and thus NO)-dependent oncogenic signaling (72). In fact, Ets-1 activation following phosphorylation by MEK/ERK—in turn, activated by SNO-Ras—resulted in the expression of basal-like markers, as P-cadherin and S100A8 to name a few (75), as well as metastasis-related proteins such as CTSB and MMP-7 (76). Since breast tumors, differently from other cancer types, more rarely harbor Ras mutations (77), the *S*-nitrosylation of Ras and thus the activation of Ets1 signaling axis may indeed explain the wild-type Ras-mediated tumorigenesis of cancers overexpressing NOS2.

### Protein stability

*S*-nitrosylation can also directly affect protein stability and turnover in different ways, including cases in which protein stability and/or the propensity for proteasomal degradation is enhanced or reduced upon the *S*-nitrosylation of target cysteines.

An example is the caveolin-1 (Cav-1) protein that is enriched in 50–100 nm sized cell membrane invaginations with a structural role. These invaginations are implicated in many cellular pathways, such as endocytosis, lipid homeostasis, and signal transduction (78). Cav-1 has been described as having a controversial role in cancer development, with pro- and anti-tumorigenic effect depending on the context and the specific cancer type (79, 80). Interestingly, a relation between Cav-1 and NO has emerged in the context of anoikis - i.e., the detachment-induced apoptosis—in lung cancer cells, where it has been demonstrated that Cav-1 is rapidly ubiquitinated and degraded by the proteasome after cell detachment and anoikis (81). In the same work, the authors observed that, upon exposure of the cell to NO donors, *S*-nitrosylation of Cav-1 was able to stabilize the protein—inhibiting its proteasomal degradation—pointing at a crucial role of the NO-mediated stabilization of Cav-1 in anoikis regulation.

Solid tumors are often characterized by a hypoxic (or even anoxic) microenvironment caused by scarce oxygen supply. Cancer cells activate a series of pathways promoting angiogenesis and other survival pathways to overcome hypoxia (82, 83). The master regulator of the activation response to low oxygen tensions is represented by the hypoxia-inducible factor 1 (HIF-1), a transcriptional complex made up of the constitutively expressed HIF-1β/ARNT and HIF-1α subunits, continuously synthesized, and degraded under normoxic conditions to be finally stabilized by hypoxic conditions (84). Because of this tangled role in cancer development, HIF-1 has been (and still is) at the heart of many scientific studies that have highlighted the existence of a multitude of post-transcriptional and post-translational mechanisms for regulating the activity of this protein (85). The degradation pathway of HIF-1α subunit in normoxic conditions starts with its hydroxylation by prolyl-hydroxylases (PHDs) in the oxygen-dependent degradation (ODD) domain. This event then targets the protein for ubiquitylation and the subsequent rapid degradation by the proteasome (86, 87). With regard to this, *S*-nitrosylation plays an active role in controlling the stability of the HIF-1α subunit. Indeed, in murine cancer cell line models, Li et al. (88) demonstrated that exposure to ionizing radiation stimulated NO generation in tumor-associated macrophages (TAMs). As a consequence, HIF-1α underwent *S*-nitrosylation on Cys533 (corresponding to the human Cys520), in turn inhibiting its binding with the E3 ubiquitin protein ligase part of the von Hippel-Lindau (vHL) tumor suppressor protein complex responsible of HIF-1 α degradation. This mechanism is of interest not only in relation to the already established role of HIF-1α in cancer therapy (89) but also for other diseases involving the immune system and inflammation (90).

We already mentioned the role of molecular chaperones, such as Hsp90, in cancer development and the regulation of its activity through *S*-nitrosylation (see section Influence of *S*-nitrosylation on Catalytic Activity of Enzymes). Hsp90 is highly conserved and with up to four different homologs in higher eukaryotes, such as the mitochondrial tumor necrosis factor receptor-associated protein 1 (TRAP1) (91). Recently, TRAP1 has been reported as a target of *S*-nitrosylation on its Cys501 with the effect of making the chaperone more prone to proteasomal degradation (40). In fact, levels of TRAP1 were reduced in human hepatocellular carcinoma cell lines depleted of *S*-nitrosoglutathione reductase (GSNOR), representing the best documented denitrosylase involved in regulating the levels of SNO-proteins in the cell (see section Enzymatic Regulation of *S*-nitrosylation). TRAP1 degradation was indicated being the causative event underlying the increase of succinate dehydrogenase (SDH) levels and activity. A few years ago it has been shown that TRAP1 is able to support cancer growth decreasing SDH activity, this leading to HIF-1α stabilization through the increase of succinate levels (41).

Recently, we and others showed that structural computational methods efficiently predict when mutations are likely to destabilize the protein in the context of disease-related mutations (including cancer) (92, 93, 94, 95). Our measurements correlate with the rate of proteasomal degradation (95). In principle, similar approaches could be applied to predict how *S*-nitrosylation of Cys could alter protein stability and proteasomal degradation, as well as if the effect is likely to be structurally destabilizing or not. A major bottleneck in this context is the current lack of proper parameters for this uncanonical modification (see section Physical Models for *S*-nitrosothiols). Another computational approach useful to this scope could be the estimate of solvent accessibility of the ubiquitination sites for proteasomal degradation of the target proteins on structural ensembles collected by molecular simulations of *S*-nitrosylated and unmodified protein structures. A SNO-induced propensity to faster rates of proteasomal degradation might be also associated with higher solvent accessibility of the ubiquitination sites induced by the PTM.

### Protein-protein interactions

An additional way used by *S*-nitrosylation to regulate protein function is through the control of protein-protein interactions. In the context of cancer progression, integrins represent a family of cell adhesion receptors that are attractive targets due to their ability to regulate cell morphology, cell-cell interaction, and signal transduction in the extracellular matrix (ECM). These processes are potentially linked to all the stages of tumor development (initiation, progression and, metastasis) (96). In 2012, Isaac et al. have demonstrated that in prostate cancer (PCs) cell lines several cysteines of integrin α6 (ITGα6)—a subunit of integrin α6β1, usually overexpressed in PC cells and the corresponding lymph node metastases - are targets of *S*-nitrosylation. Particularly, Cys86 *S*-nitrosylation of ITGα6 enhances its binding to ITGβ1 (overexpressed in PC cells too), decreasing the extent of cell adhesion and potentially explaining the ability of iNOS and NO to promote migration of the cancer cells (97, 98).

In conclusion, despite the fact that we illustrated separately the different strategies used by *S*-nitrosylation to impact on the cellular proteome, it is important to bear in mind that many of the processes individually discussed are however highly interconnected, as well as they can occur for the same SNO target. The relation between *S*-nitrosylation and cancer can be seen as a tangled map of connections, many of which are still poorly defined.

## Prediction and annotation of *S*-nitrosylated proteins

### The database of *S*-nitrosylated proteins: dbsno

Technical advances in mass spectrometry-based proteomics have improved the identification of *S*-nitrosylation sites (14, 99–103) and contributed overcoming the challenges due to the labile and highly dynamic nature of the thiol redox forms. This has been made possible by the availability of chemically-selective approaches to detect thiol redox modifications in concert with mass spectrometry-based proteomics (103).

These advances made now available a large list of experimentally identified SNO sites that require proper annotations and curations in publicly available repositories.

dbSNO2.0 (http://dbSNO.mbc.nctu.edu.tw) has been developed as a freely available resource to collect and explore the SNO sites of experimentally-identified *S*-nitrosylated proteins (104, 105). Moreover, regulatory networks are annotated for the *S*-nitrosylated proteins in the database, along with annotation of relevance in disease. dbSNO is the first manually curated repository for SNO peptides accounting for more than 4,000 SNO sites in almost 3,000 proteins. Annotation on targets with known 3D structure are also included together with information on solvent accessibility, neighboring residues and side-chain orientation for up to 298 substrate cysteine residues.

dbSNO2.0 can provide a useful framework for the redox biology community thanks to its availability of structural annotation for the computational and modeling studies on *S*-nitrosylated proteins. The bottleneck will be a regular update and curation of this research. We could expect that the number of characterized SNO sites will grow at a fast rate if we consider that the computational analyses of annotated protein databases using predicted SNO motifs indicate that up to 70% of the proteome may be targeted by *S*-nitrosylation (see section Prediction of *S*-nitrosylated Targets and SNO Sites). In the future, the SNO community could benefit of more collaborative and interdisciplinary efforts to develop and maintain a broader up-to-date repository and where also the experimental datasets from SNO-proteomics can be deposited for re-analysis, inspired by the efforts that the genomic community already established.

### Prediction of *S*-nitrosylated targets and sno sites

In the last few years, as the topic of *S*-nitrosylation gained importance and recognition among researchers, several attempts have been made to predict *S*-nitrosylation sites on proteins based on experimental data as a reference, using machine-learning algorithms on protein sequence data. Nonetheless, predicting SNO-sites has proven challenging for a number of reasons. First of all, a simple sequence linear motif that clearly identifies *S*-nitrosylation sites does not exist, as detailed in section Computational Structural and Chemical Studies of *S*-nitrosylation. The chemical-physical features of the environment surrounding the SNO site are crucial determinants for SNO specificity, and the pK_a_ of the thiol moiety of a cysteine greatly influences its propensity to be *S*-nitrosylated. Accurate methods for the prediction of pK_a_ from the 3D structure of proteins are currently available, such as PROPKA (106). Thus, the prediction of cysteine pK_a_ would be in principle a viable alternative when the 3D structure is available, or, even better, when an ensemble is available and protein flexibility can be taken into account (107). However, an analysis of structural determinants for *S*-nitrosylation showed that, at least in a limited data set of 55 proteins, features such as the acid–base motif flanking NO-Cys, hydrophobic content, predicted pK_a_ and solvent accessibility do not distinguish SNO-sites from non-*S*-nitrosylable cysteines (108). This study also reports that the presence of a SNO-site is correlated with the presence in the surrounding area of an acid-base motif, possibly with different functions than the mere activation of Cys or stabilization of SNO, such as in facilitating protein-protein interactions that would, in turn, induce *S*-nitrosylation. These results indicate that, while the local chemical environment around the SNO sites is certainly influencing reaction rates, predicting *S*-nitrosylation could benefit from a less reductionist approach.

Despite what just described, machine learning approaches tested so far have used protein sequences and sequence-derived features only; furthermore, even considering these, the set of experimental data on which machine algorithms can be trained is limited, at least respect to other PTMs such as phosphorylation, and typically unbalanced. This problem has been partially relieved by the publication of curated collections of SNO sites, such as the dbSNO database (105) and other manually curated datasets, as for example those by Xue et al. (109) and Li et al. (110). These resources do not alleviate the issue of using sequence data exclusively. Approaches based on protein structure would probably be more viable or at least include features that are more relevant to the problem at hand.

Given the limitations above, it is not surprising that the performance of SNO-site predictors has been so far consistently lower than predictors for other types of PTMs, with a Matthews correlation coefficient typically between 0.2 and 0.4. It should be emphasized, however, that no systematic review of performance on a common and independent test set has been performed so far. The fact that some of the methods have not been released as implementation or source code and that some of the web servers for SNO-site prediction are unreachable at the time of writing also makes fair comparisons difficult.

The different approaches tested so far for the prediction of SNO-sites are summarized in Table 1. Most of them relied on machine learning algorithms such as Support Vector Machines or (SVM) k-Nearest Neighbours (kNN) to classify Cys residues as *S*-nitrosylation sites or not. As feature space, all the methods considered amino-acid composition of the residues flanking the putative SNO-cysteine, often together with more structurally-oriented predictions such as physicochemical properties of amino acids, predicted secondary structure, predicted disorder propensity. This led, in turn, to a relatively high number of features among which the most discriminating and uncorrelated ones are selected, ranking them using methods as the minimal redundancy maximal relevance (mRMR) or the relative entropy selection. From a historical perspective, computational prediction of SNO sites has been tackled since 2006, when Hao et al. trained a support vector machines (SVM) classifier on a small dataset of SNO-proteins experimental data. The model turned out to barely outperform random chance (111). The first released algorithm designed to classify cysteine residues is named GPS-SNO (Group-based prediction system) (109), which uses an optimized scoring function to discriminate between SNO and not-SNO sites. Another prediction tool named SNOSite (112) was released in 2011, based on a collection of SVM models, each derived from sequences clustered according to a maximal dependence decomposition scheme to obtain statistically significant conserved motifs. CPR-SNO used a SVM classifier in which different encoding schemes for the protein sequence flanking the potential SNO-Cys were tested (113). In 2012, Li et al. (110) used the minimal-redundancy maximal- relevance (mRMR) method to determine the importance of several features of the sequence flanking C-sites to predict SNO-sites. The features included amino acid type, physicochemical features and conservation, disorder and secondary-structure propensity as well as solvent accessibility. Incremental feature selection was used to determine the set of features that would give the best performance. In 2013, two different predictors by Xu and colleagues were also released. The first, iSNO-PseACC (114) uses position-specific amino acid propensity into the form of pseudo amino acid composition with conditional random field models to predict the presence of SNO-sites, while the second, iSNO-AAPair (115) takes into account the coupling effects between residues close in sequence. In 2014, a paper published by Zhang et al. (116) used a scheme similar to Li et al. (110), in which a subset of features was calculated from the flanking sequence, feature selection was operated on them using relative entropy selection and incremental feature selection, while classification was done through the kNN algorithm. The final model was made available through the PSNO web server. In 2014 again, a paper by Huang et al. (117) used a similar approach to Li et al. (110) which also uses the same feature set, using the kernel sparse representation classification together with mRMR. Finally, another method has been implemented in iSNO-ANBPB and released in 2014, which uses an adapted normal distribution bi-profile Bayes (ANBPB) for feature extraction together with SVM (118).

**Table 1:** Summary of current methods for prediction of *S*-nitrosylation.

**Name**	**Year**	**Availability**	**Link**	**Number of citations (Google scholar, April 2018)**	**PMID**
GPS-SNO	2010	Webserver, standalone	http://sno.biocuckoo.org/	111	20585580
SNOSite	2011	Webserver	http://csb.cse.yzu.edu.tw/SNOSite/Prediction.html	54	21789187
CPR-SNO	2011	Webserver	http://math.cau.edu.cn/CPR-SNO/CPR-SNO.html^a^	21	21271979
–	2012	None	None	54	22178444
iSNO-PseAAC	2013	Webserver	http://app.aporc.org/iSNO-PseAAC/	112	23409062
iSNO-AAPair	2013	Webserver	http://app.aporc.org/iSNO-AAPair/	113	24109555
PSNO	2014	Webserver	http://59.73.198.144:8088/PSNO^a^	42	24968264
–	2014	None	None	5	25184139
iSNO-ANBPB	2014	None	None	41	24918295

a *Link to the website not working at the time we wrote this review article*.

## Computational structural and chemical studies of *S*-nitrosylation

### *S*-nitrosylation sites specificity

Many mechanisms can lead to the *S*-nitrosylation of proteins: reaction with NO_2_, S-transnitrosylation, thyil radical recombination, and transition metal catalyzed pathways (see also sections *S*-nitrosylation of Cysteine Residues and Enzymatic Regulation of *S*-nitrosylation). These different pathways do not necessarily take place in the same cellular and protein environment. Thus, it is especially difficult to describe the microenvironment properties driving the specificity of *S*-nitrosylation sites (4, 14, 119, 120).

Albeit no systematic environment features have been found to be predictive descriptors for this PTM, the structural microenvironment would favor the susceptibility of cysteines to react with NO or undergo *S*-transnitrosylation. However, one has to keep in mind that these features could also favor cysteine reactivity toward other PTMs, hence it is really difficult to depict a precise panel of conditions that have to be fulfilled for *S*-nitrosylation to take place (4, 119).

Intensive investigations on the subject led to the conclusion that several environment features might have an influence on the specificity of cysteine targeting mechanisms associated to *S*-nitrosylation. The latter are certain characteristics that are subtler than a simple primary sequence effect, so that no discernable consensus sequence to accurately predict Cys SNO sites has been found so far (14, 28, 111, 121). In the 90's, Stamler and coworkers were the first to report the role of basic and acidic side-chains in the proximity of a cysteine in promoting *S*-nitrosylation, highlighted by their work on the *S*-nitrosylated hemoglobin protein (122, 123). In Stamler's model, a histidine residue proximal to the Cysβ93 in the oxygenated R-state of hemoglobin facilitates a base-catalyzed *S*-nitrosylation whereas a proximal aspartic acid in the deoxygenated T-state favors acid-catalyzed denitrosylation, coupling the oxygenation status of the hemoglobin to the SNO formation and release during the respiratory cycle. In another work, the acid-base motif in SNO-proteins has been expanded to include residues −6 and +6 from the target Cys and the notion of hydrophobic regions surrounding the SNO-Cys (121).

Acid-base motifs have been also reported at the tertiary structure level, i.e., not as short-linear motifs in the sequence space. In this case, acidic and basic residues in the proximity of the SNO site are contributed by different regions of the protein that are brought together in the 3D structure, as first found in the methionine adenosyltransferase (MAT) protein (124). The *S*-nitrosylation of the Cys121 of MAT is indeed promoted by two arginines which are distal in the primary sequence but close in the tertiary structure and lower the Cys pKa. Similarly, 3D acid-base motifs have been found in the case of caspase-3 and aquaporin-1 proteins (12).

Since these first works on hemoglobin and MAT, several data consolidated the hypothesis of a 3D acid-base motif (12, 108, 125–129). Overall, the charged acidic and basic side chains in proximity of the SNO site regulate *S*-nitrosylation by influencing both thiol pKa (and as a consequence their reactivity) and SNO stability. As a general rule, the presence of an acid-base motif within 8Å of the target cysteine could facilitate interactions that make it to *S*-nitrosylation—e.g., protein-protein interactions in the case of *S*-transnitrosylation (130–132).

Besides the acid-base motif, other structural characteristics appeared to drive the specificity of cysteine reactivity toward CysNO. For instance, the presence of hydrophobic residues in the surroundings of the *S*-nitrosylation sites has also been proposed. As a matter of fact, *S*-transnitrosylation and NO oxidation reactions may preferentially take place on cysteine residues located in a hydrophobic pocket because of their low pK_a_ that, in turn, make them prone to react (10, 131–138). Hydrophobic environments favoring *S*-nitrosylation are not limited to hydrophobic residues in the protein target. Indeed, they can also be contributed by membranes or interactions with other proteins (12).

This specific environment may also favor interactions with transition metal nitrosyls (127, 128, 139), as well as stabilize radical nitrosating species in the case of thiyl radical recombination (128, 140). Interestingly, studies suggested that acid-base motifs would be correlated with location of the target cysteines on α-helices, whereas hydrophobic environments would be more associated with *S*-nitrosylation on Cys sites located in β-sheets (130). Further factors that govern *S*-nitrosylation specificity are steric hindrance and solvent accessibility, that have also been proven to be key-features for *S*-nitrosylation promotion (3, 127). These two determinants are especially important in the case of *S*-transnitrosylation, in which protein-protein interactions are crucial. However, bioinformatic investigations revealed that, surprisingly, around 50% of the *S*-nitrosylation and *S*-glutathionylation sites are actually deeply buried in the native structure of the protein (141). Several studies revealed that buried cysteines may still be susceptible to undergo *S*-nitrosylation when surrounded by hydrophobic residues, by channeling of NO to target thiols (6, 10, 131, 133–138). This might be the case of the recent buried SNO site discovered in c-Src kinase where *S*-nitrosylation has a clear effect on protein activity and relevance in a cancer context (see section Computational Structural and Chemical Studies of *S*-nitrosylation). NO oxidation might also be favored upon co-localization of the target protein with NOS isoforms (3, 119), indeed enhancement of *S*-nitrosylation has been observed especially in the presence of surrounding eNOS in the Golgi apparatus (17, 142, 143). Compartmentalization of NO is induced by its quick consumption after production (< 0.1 s) through reaction with molecules that colocalize with NOS (17). Furthermore, several NOS and NOS-interacting proteins are well-known to undergo *S*-nitrosylation—e.g., S100A8/A9, Dlg-4 and Cav-1. Thus, co-compartmentalization of the target protein with NOS could be considered as one of the many factors driving *S*-nitrosylation specificity, with the nitrosyl group being transported to a distant cellular zone through *S*-transnitrosylation.

In the analysis of cysteine SNO sites, we should also take into account the capability of the cysteine side chain to be involved in hydrogen bonds and how the substitution of the SH group with SNO can affect the native hydrogen bond network. Cys can serve as a hydrogen bond (HB) donor when protonated (SH) as well as a HB acceptor in both protonated and deprotonated states (S^−^) (144).

Hence, acid-base motif and hydrophobic pocket proximity, appropriate redox potential, solvent accessibility, steric hindrance and colocalization with NOS have been highlighted as significant factors driving *S*-nitrosylation site specificity. Based on these considerations, machine learning techniques have been used in order to investigate *S*-nitrosylation sites. Several web servers have been designed to predict cysteines susceptible to be *S*-nitrosylated based on the protein sequence, and the dbSNO database is also available online to probe CysNO environment in PDB entries and predict potential SNO-proteins that may play a role in NO signal regulation in cancer cells - see details in section Prediction and Annotation of *S*-nitrosylated proteins.

### SNO-induced long range and allosteric effects

Little is known about the potential distal effects induced by *S*-nitrosylation. Nevertheless, allostery has been proposed to be an important factor for the promotion of *S*-nitrosylation/denitrosylation (128), and allosteric effects due to *S*-nitrosylation have been observed to play an important role in the regulation of the activity of several proteins (97, 145, 146, 147). Allosteric SNO-induced effects have been also postulated for the denitrosylase GSNOR, pointing out an intriguing feedback regulatory mechanism of NO signaling (see sections GSNOR System and GSNOR in Cancer).

Another striking example is the inactivation of the inducible nitric-oxide synthase (iNOS) upon auto-*S*-nitrosylation. NOSs (iNOS, eNOS, and nNOS) are well known to work as homodimers (148), with—at the interface between the two monomers—a zinc atom coordinated to four cysteines in a conserved ZnS_4_ motif (149, 150). The latter is of utmost importance for the protein-protein interactions and the integrity of the homodimer, hence its activity. Therefore, the regulatory effects linked to the allosteric disruption of the interactions at the dimer interface have been extensively investigated for the design of new NOSs ZnS_4_-related therapeutics (151–155). Interestingly, this site is specifically *S*-nitrosylated at the Cys109 position in iNOS. The latter induces the release of Zn^2+^, coupled to a strong destabilization of the dimer, resulting in its disruption and iNOS inactivation (145). Upon high NO concentration, *S*-nitrosylation of iNOS limits its activity (i.e., NO production) through an allosteric mechanism driving the dimer/monomer balance (145, 156). Thus, the Cys109 specific *S*-nitrosylation plays an important role in the regulation of iNOS activity, whose dysfunction has been shown to play an important role in tumor growth in several cancer types (157). Noteworthy, eNOS dimer dissociation upon treatment with NO donors has also been reported (158), suggesting a similar equivalent autoinhibitory mechanism. Similar structural impact, although less pronounced, has been reported for the NMDA receptor, for which *S*-nitrosylation has been suggested to allosterically regulate the ligand-binding, regulatory and linker regions (146, 159, 160). Likewise, the cyclic nucleotide-gated channels (CNGcs), a class of ionic channels that permeate cations and are especially important in sensory-receptor cells, might be allosterically regulated by *S*-nitrosylation of a specific cysteine located in their ligand-binding region (147).

Besides, relatively small distal effects of *S*-nitrosylation have been observed in certain X-ray structures of SNO-proteins (161–164). This behavior nicely fits in the context of allosteric effects that can occur without a marked change in protein shape, as attested in many other biological cases (165, 33). For instance, hemoglobin (Hb) *S*-nitrosylation leads to local reorganization but no large changes in the quaternary structure of the tetramer in the crystals (162). However, the authors suggested that larger effects might occur in solution, with a shift in the balance between Hb R and R2 states. In a similar fashion, S100A1 *S*-nitrosylation leads to distal reorganizations of the linker region and the two helices III and IV from the C-terminal EF-hand, that are known to be important for target recognition (164).

Nevertheless, the paucity of experimental structures of SNO-proteins does not allow to efficiently probe the distal effects that could be induced by *S*-nitrosylation. In this context, methods based on MD simulations using reliable force field parameters for Cys-NO would constitute a considerable asset to explore such reorganizations. Indeed, many methods to study distal conformational changes, including methods based on MD-derived ensemble, have been proposed and successfully applied to the study of long-range effects induced by protein PTMs such as phosphorylation or other perturbation of the protein native structure (32–35, 166–168) and they can be naturally translated to the study of allosteric effects promoted by *S*-nitrosylation.

### A case study: effects of *S*-nitrosylation on Src kinases

Kinases are usual suspects in the context of cancer research (169, 170, 171). Intriguingly, *S*-nitrosylation has been recently pointed out as regulatory mechanisms for kinases (141, 172), which are enzymes with the main regulatory role for another PTM, i.e., phosphorylation, highlighting an interesting cross-talk between different PTM cellular signals.

As mentioned in the previous sections, Src kinases are a family of kinases responsible for cellular proliferation, differentiation and survival (172). Disregulation of Src kinases has been linked to different cancer types. They are multi-domain proteins, including four different domains of which one carries out is the catalytic activity. Two tyrosines (Tyr416 and 527) are regulated by phosphorylation and their phosphorylation is responsible of either activation or deactivation of the kinase, respectively.

Apart from phosphorylation, another layer of post-translational regulation—relying on *S*-nitrosylation—has been demonstrated for the c-Src kinase. The SNO site is the Cys498, which is one of the nine cysteines of the human Src kinase (47). c-Src is known to promote cancer cell invasion and metastasis and its *S*-nitrosylation enhances the protein activity but the structural mechanisms behind this have been poorly understood. Cys498 is also conserved in other kinases of the Src family, suggesting a common mechanism, as attested by the fact that the c-Yes kinase activation is also mediated by NO (47). These data overall link NO-dependent activation of c-Src to cancer cell invasion and metastasis but the structural and molecular details are still elusive.

In a recent computational work, Rando and coworkers applied different computational structural analyses accounting for electrostatic, steric and hydrophobic properties to compare the Cys498 selective SNO site with the other three c-Src cysteines that are not affected by *S*-nitrosylation. Their data pointed in the direction of a rather buried and highly nucleophilic Cys with a highly hydrophobic environment in which NO can be more prone to undergo decomposition into the electrophilic intermediates (173).

As a future direction, structural studies to assess conformational changes in the *S*-nitrosylated and non-*S*-nitrosylated protein could shed new light on the NO-mediated activation of c-Src and other similar kinases, as well as properly assess the accessibility of the SNO site in the native conformational ensemble of the protein. Also, in this context, the main limitation is related to the poor availability of force field parameters to describe SNO proteins.

### Physical models for *S*-nitrosothiols (RSNOs)

The impact of *S*-nitrosylation on protein structure, function and stability can be different from one protein to the other (see section Biological Mechanisms Promoted by *S*-nitrosylation). Therefore, one could not describe a systematic structural and reactivity behavior of *S*-nitrosylated proteins. There is a real need of investigations by both experimental and theoretical means, which represent a colossal yet of utmost importance work with the aim at gaining knowledge about the important phenomena driving RSNOs formation and reactivity.

*S*-nitrosothiols exhibit a highly complex chemistry, which represents a real challenge for theoretical studies. In the last decade, efforts have been dedicated to the development of an accurate structural and electronic description of the -SNO group, by both experimental and theoretical means (174–176). Concerning theoretical investigations, a certain amount of high-level quantum chemistry studies has been performed on small RSNOs (mainly with R = H,Me). The main part of the theoretical studies focused on the description of the thionitrous acid HSNO, the smallest RSNO (128, 143, 177–180). Indeed, the high complexity of the -SNO moiety requires the use of time-demanding calculations. Thus, the size of the system is rapidly limited by the computational resources. The nature of the S-N bond is especially challenging to investigate, and lots of efforts have been made to gain information about the properties of this bond. Values of the bond dissociation energy (BDE) and the activation energy corresponding to the transition between the *cis* and *trans* isomers have been computed using diverse levels of theory (181–184). One of the most recent computational studies using high level CCSD(T) coupled to CBS extrapolation methods (179) suggested that the *cis*- *trans* interconversion requires an activation energy up to ~9 kcal/mol, with the *cis* isomer slightly less stable than the *trans* one, roughly by 0.1 kcal/mol. Investigations on larger RSNOs moieties have shown that the nature of R may have a strong influence on RSNO chemistry, hence CysNO structure might exhibit different features than HSNO (128, 185–189). However, there is still a lack of theoretical studies about CysNO properties, with only few investigations being reported concerning models structurally closer to the *S*-nitrosylated cysteine than HSNO.

Electronic properties of the -SNO group have been highly investigated by computational means. Especially, a lot of work is available on HSNO structure description by high-level QM methods and even Car-Parrinello metadynamics (179, 181, 184, 190–192), which shed light on the multi-reference character of the -SNO group. Indeed, the complexity of the S-N bond nature might result from the combination of three different resonance structures: the neutral -S-N = O, the zwitterionic -S^+^ = N-O^−^, and the RS^−^/NO^+^ ion pair. Further investigations on the larger CH_3_SNO model confirmed this feature, yet it seems less pronounced for CH_3_SNO than HSNO (186). Unfortunately, no further evidences about RSNOs multi-reference character has been reported for larger models of RSNOs, the computational cost of such high-level calculations being prohibitive.

Nevertheless, these investigations underlined the difficulty to obtain an accurate description of *S*-nitrosothiols structure, with the S-N bond exhibiting very complex chemical properties. Hence, one should pay a particular attention while dealing with such systems, by starting with a careful choice of the level of theory.

The unusual electronic structure of RSNO and its multi-reference character makes it a difficult system to accurately model. Hence, high level *ab initio* QM methods should be used in order to obtain a reliable and accurate description of the complex electronic density of the SNO moiety. However, the use of such quantum calculations is computationally very demanding when it comes to study models larger than HSNO or CH_3_SNO. Thus, benchmark studies have been led to assess the capacity of the less time-consuming DFT and TD-DFT methods to reproduce structural and electronic features obtained by high level *ab initio* methods and experiments. Overall, several of these theoretical investigations highlighted the reliability and robustness of the B3P86 functional with large basis sets for the calculation of S-N bond energy dissociation, spectroscopic and structural properties of small RSNO (182, 189, 193). Likewise, the PBE0 functional with large basis sets has also been revealed as a good compromise between computational time and accuracy in describing RSNO properties (179, 186, 188, 194). However, it is always strongly recommended to verify results obtained by DFT methods using more computationally demanding higher-level methods (186). Likewise, experimental data on RSNOs characteristics, mainly from NMR studies, have been reported and can validate values predicted by computational chemistry (44, 195–197).

The electronic and structural properties of RSNO are highly modulated by the molecular environment, especially in the presence of proximal charges (e.g., upon coordination to metal ions), with large fluctuations of the S-N bond stability being observed in several experimental and theoretical works (182, 185, 188, 198, 199). Studies of RSNO interacting with metals especially highlighted the dramatic influence of surroundings on the S-N bond nature. For instance, coordination to Cu^I^, which is known to play important roles in NO-release regulation by catalyzing the decomposition of RSNOs, tends to weaken the S-N bond upon S-coordination while N-coordination was predicted to strengthen it (182, 200).

**Figure 4:**
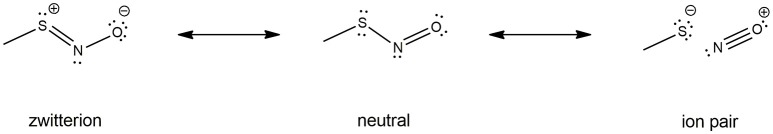
RSNO resonance structures, balanced between the neutral **(center)**, the zwitterion **(left)** and the ion pair **(right)** forms. The *trans* conformer is depicted here, but the *cis* is also possible though less stable, as mentioned in section Physical Models for S-nitrosothiols (RSNOs). The neutral form is the most abundant one, the other ones being only minor conformations with dramatically opposite features—hence the dual reactivity of RSNOs with nucleophiles. The relative abundance of the three RSNO forms is highly depend on its microenvironment and the nature of the R group. For instance, the neutral/zwitterion/ion pair ratio is 79/11/10% vs. 75/15/10% for HSNO and CH_3_SNO respectively.

An interesting property of the -SNO moiety is the multi-reference character induced by the dramatic difference between its resonance structures (Figure 4), as highlighted in several theoretical works published by Timerghazin's group (184–186, 188, 201). Indeed, the use of an external electric field (EEF) in QM calculations brought out the high polarizability of the–SNO moiety, with modulation between two minor resonance structures, which exhibit opposite charge distribution and reactivity. The first one exhibits an ionic structure, with the charge located mainly on the sulfur atom, interacting with the electrophilic NO^+^ moiety through a long and weak S–N bond. Upon opposite polarization of the EEF, the -SNO adopts a totally different configuration, with the charge located on the NO double bond, the sulfur atom being in this case prone to undergo nucleophilic attacks. The balance between the zwitterionic, neutral, or ionic conformations might thus be highly influenced by the electric field induced by the -SNO chemical environment and lead to a fluctuating reactivity of this moiety (185). The coexistence of such antagonist structures brings explanation to the contradictory observations that have been published concerning RSNO structure and reactivity (193, 202–205).

Nowadays the computational resources available do not allow to model the dynamics of an entire protein at the QM level of theory. Thus, investigations about the *S*-nitrosylated cysteine electronic structure have been performed on reduced models only. The largest one reported so far being the portion of an α-helix *S*-nitrosylated *in silico*, on which Talipov et al. performed QM/MM calculations to probe the effect of proximal charged amino acids on CysNO electronic structure (188). Nevertheless, QM/MM calculations are way too time-consuming to be systematically used to investigate influence of the protein environment in realistic models, since the dynamics of the system might also have a strong influence on the cysteine environment. Furthermore, Talipov et al. suggested that even small allosteric effects could induce dramatic effects on the CysNO reactivity. Thus, the dynamics of the system should be taken into account when studying the *S*-nitrosylated cysteine behavior in a protein environment. The use of all-atom MM-MD simulations can help toward this goal. However, attention should be devoted to the choice of the force field parameters used to describe the highly complex *S*-nitrosylated cysteine structure.

So far, AMBER and GROMOS force field parameters have been developed to describe CysNO and few case studies of proteins harboring CysNO by classical molecular dynamics simulations have been reported. On the one hand, the AMBER parameters developed by Han et al. have been generated using quantum mechanics (206) and validated using as a model system a *S*-nitrosylated thioredoxin crystal structure on Cys69 (207). On the other hand, Petrov et al. (208) parameterized CysNO by deriving GROMOS force field values. Moreover, online tools have been developed to insert PTMs *in silico* using GROMOS force field parameters, including the cysteine *S*-nitrosylation: the Vienna-PTM and the Automated Topology Builder (ATB) servers (209–211). Nevertheless, an in-depth look into the values of these two different sets of parameters highlights not negligible differences (Tables 2, 3). Thus, these parameters have to be extensively validated against experimental data to assess their efficiency in describing a such complex chemical structure. The major bottleneck in this field is related to the fact that only a minority of experimental structures is available by NMR and X-Ray crystallography (161–164, 207, 212–216) and classical MD simulations using reliable force fields usually provide predictive data about structural and dynamical behavior of biosystems. However, in the CysNO case, it would be difficult to model with standard force fields using point charges, given that the polarization of the—SNO moiety is likely to undergo a dramatic variation of the reactivity depending on its micro-environment. To overcome this issue, one might consider to use polarizable force fields, which are undergoing marked improvements (217).

**Table 2:** Comparison between GROMOS and AMBER parameters for *S*-nitrosylated cysteine with regards to atomic charges.

	**GROMOS**	**AMBER**
Atomic charges (S)	0.1	−0.0735
Atomic charges (N)	0.35	0.0355
Atomic charges (O)	−0.45	−0.1522

**Table 3:** Comparison between GROMOS and AMBER force field (FF) parameters for *S*-nitrosylated cysteine (i.e., bonds, angles, dihedral angles).

	**Bonds**			**Angles**		**Dihedral angle**
	**C-S**	**S-N**	**N-O**	**C-S-N**	**S-N-O**	**C-S-N-O**
**FF NAME**						
GROMOS	gb_31	gb_31	gb_5	ga_30	ga_26	gd_21
AMBER	CT SH	SH NC	NC O	CT SH NC	SH NC O	CT SH NC O
**EQUILIBRIUM DISTANCE/ANGLE**						
GROMOS	0.178	0.178	0.123	121	120	0
AMBER	0.181	0.1755	0.1165	103.96	116.58	180
**FORCE CONSTANT (kJ mol**^−1^ **nm**^−1^**)**						
GROMOS	5.94E+06	5.94E+06	1.66E+07	685	530	16.7
AMBER	1.98E+05	2.77E+05	6.61E+05	354.8	527.37	22.51
**PHASE**						
GROMOS	/	/	/	/	/	2
AMBER	/	/	/	/	/	2

Overall, investigations that have been led so far on small RSNO models provide a solid basis toward the understanding of *S*-nitrosylated cysteines structural properties within a protein environment. The system size and the simulation time scale are known to be very rapidly prohibitive while using QM and hybrid QM/MM (-MD) methods. Besides, classical MD simulations are nowadays a method of choice for the theoretical investigation of dynamics of proteins and macro-molecules in general (32, 33, 218), but its reliability relies on the force field accuracy. Hence, a particular effort should be realized in order to bypass limitations of standard force fields and find a sustainable solution in order to unravel the complex mechanisms underlying proteins *S*-nitrosylation.

## Reactivity of *S*-nitrosothiols

**Figure 5:**
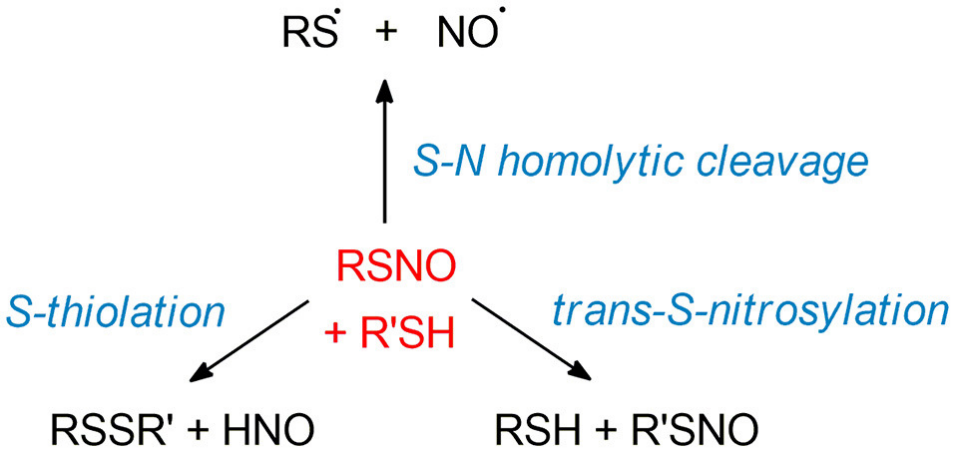
SNO reactivity. We here illustrated the main reactions involving RSNOs. **Top:** homolytic cleavage of the S-N bond, consisting in the loss of nitric oxide with a remaining thyil radical species. **Left:** *S*-thiolation between RSNO and a R'SH thiol, leading to the formation of a disulfide bridge RSSR' and a nitroxyl moiety HNO. **Right:** *S*-transnitrosylation process inducing the transfer of NO from RSNO to a R'SH thiol. The detailed mechanisms of these reactions are still poorly understood and thus not depicted here.

The unusual structure of *S*-nitrosothiols (RSNOs), balancing between three different mesomeric forms, implies a complex chemistry of these moieties. The biological relevance of RSNOs relies on their important role in NO storage and transport, as well as their function as HNO donors *in vivo* (219–224). As RSNOs are relevant to a broad spectrum of diseases, ranging from asthma to cancer (6, 8, 140, 225–227), extensive works have been reported with the final goal of developing new SNO-related therapeutical strategies. Efforts have been made especially for the design of new RSNO-inspired NO-releasing biomaterials (228–230). In this framework, a deep knowledge of the mechanisms driving the RSNOs reactivity is of utmost importance for the design of therapeutics, with implications for the treatment of a large range of diseases. Theoretical investigations have been led in order to unveil the electronic mechanisms ruling RSNO denitrosylation. The latter can take place through several reaction pathways: *S*-transnitrosylation, disulfide bridges formation (*S*-thiolation), and homolytic cleavage of the S-N bond—see Figure 5.

### RSNO-thiol interactions

Albeit the *S*-transnitrosylation and *S*-thiolation have been studied extensively by experimental means (22, 26, 28, 131, 231, 232), there is only a little amount of theoretical data available. A proper electronic description of the *S*-nitrosylated cysteine requires the use of high-level quantum methods, whereas the current computational resources do not allow to study such a reaction in a protein model, meaning that current studies are limited to small RSNO models. Moreover, only one work has been published about the electronic mechanisms driving *S*-thiolation by Ivanova et al. using MeSNO and MeSH models (194). According to their DFT calculations on this simplified model system, the reaction takes place in three phases. First, the thiol proton is transferred to the—SNO nitrogen atom, leading to a thiolate MeS^−^ and the MeSNHO^+^ moiety. The latter undergoes a strong delocalization of the electronic density on the oxygen atom, increasing the electrophilicity of the sulfur group. Meanwhile, the thiol sulfur moves out of plane to initiate the nucleophilic attack on the -SNO sulfur atom. The second phase is the formation of the S-S bond, which results in a zwitterionic species involving a tri-coordinated sulfur. The third and last phase consists in the release of HNO following the S-N bond cleavage. A similar reaction mechanism has been proposed by Moran et al. (233) for the hydrolysis of O-protonated RSNO.

The addition of explicit water molecules in the model system highlighted the possibility of a reaction assistance, with an activation energy decrease up to 20 kcal/mol. The water molecules participate to the proton transfer, but also have a stabilizing effect on the charge-separated intermediates. This stabilization is even more pronounced when moving from gas phase to a polarizable aqueous environment by using an implicit solvent model, suggesting that the environment provided by the protein embedding might also offer a similar stabilization. This hypothesis is supported by investigations highlighting the effect of external electric fields on the RSNO electronic density, that can lead to its dramatic polarization (185, 186, 188). Noteworthy, the biologically relevant competition between the *S*-thiolation and the *S*-transnitrosylation processes might then be driven by the polarization of the -SNO by its surroundings. The ionic conformation would favor thiolate attack on the NO^+^ (i.e., *S*-transnitrosylation) while its antagonist structure might be prone to undergo nucleophilic attack of thiolate on the sulfur atom with release of a HNO molecule—i.e., *S*-thiolation and disulfide bridges formation (188). Very recently, a work by Wolhuter et al. suggested that *S*-nitrosothiols could be reactive intermediates leading to disulfide bridges formation and *S*-thiolation (234). Their work highlighted the strong propensity of *S*-nitrosothiols to induce disulfide bond formation, pointing out the larger instability of *S*-nitrosothiols compared to disulfides. According to their results, *S*-nitrosothiols might be only transient moieties favoring the formation of S-S bonds, rather than the putative stable redox regulators. The study of cysteines nitrosothiols reactivity, involved in the promotion of disulfide bridges formation, is of major importance for the understanding of the complex mechanisms ruling the cell redox regulation. We thus envision that further theoretical and experimental efforts are still necessary to strengthen our knowledge in this area.

### Homolytic cleavage of RSNO

Theoretical and experimental investigations about the *S*-nitrosothiols S-N bond dissociation stressed out the ease of this reaction, both thermal, and photochemical decomposition happening at room temperature (199, 235–240). The latter reaction induces the homolytic loss of NO, eventually leading to the formation of a stable S-S bond, which involves the reaction between two newly formed thyil moieties. The weakness of the S-N bond (values from 15 to 35 kcal/mol) (184, 186, 189, 193, 203, 219, 241) may favor its rapid decomposition, although the *S*-nitrosothiol chemical environment can strongly enhance its half-life (198, 199, 204).

Recent theoretical work about the S-N photo-dissociation in model compounds (small RSNOs) shed light on the barrierless character of the process upon irradiation in the visible and UV regions as well as upon exogenous photosensitization, with population of S_1_, S_2_, and T_1_ states respectively (189). Experimental investigations showed that irradiation of *S*-nitrosothiols at UV-visible wavelengths induces the release of nitric oxide and a thyil radical (236, 242). Likewise, photo-decomposition of RSNOs has been observed in human skin upon UVA irradiation (243). Similar wavelengths have been reported for light-induced release of NO from NO-metal complexes (mostly iron, but also Mb, Co, or Ru among others), which has been widely investigated for its biological relevance and has been observed in the UV-vis-NIR regions depending on the nature of the complex (244–247).

Thermal decomposition of small RSNOs has also been investigated using high-level computational methods (184, 186, 201). In the most recent of them, Khomyakov and Timerghazin underlined the difficulty to obtain accurate values of the S-N bond dissociation energy, the convergence of the S-N bond features being especially slow due to the unusual, multi-reference character of the -SNO moiety (186). Their calculations suggested a value of 32.2 kcal/mol for the CH_3_SNO model S-N bond cleavage in implicit aqueous solvent, with a very small stabilization of energy when shifting from water to the non-polar diethylether solvent—often used to mimic the protein environment. Noteworthy, metal ions and especially copper ions can efficiently catalyze the S-N bond thermo-dissociation, making it an ultrafast process (182, 236, 248, 249).

NO transport and storage are mainly provided by iron-nitrosyl (e.g., in myoglobin and hemoglobin) and nitrosothiols compounds. These processes are known to be of utmost importance for the modulation of important cellular mechanisms such as the mitochondrial respiration (250) or vasodilation/cardioprotection (251). As mentioned previously in this review, nitric oxide is also known to have a multifaceted role in cancer biology, with both tumor-suppressing and tumor-promoting effects reported (252, 253). The redox chemistry of *S*-nitrosothiols is as rich as complex and is related to a large variety of biochemical processes. It is notably balanced by pH, temperature, UV-vis irradiations, RSNO micro-environment (influencing its pK_a_), chemical nature of RSNO, and presence of reactive compounds such as metal ions (254, 255). The dysregulation of NO flux in the tumor micro-environment has also been found to modulate the redox signaling pathways during cancer progression (256), which might consequently influence RSNOs reactivity. Several compounds are known to react with *S*-nitrosothiols such as phosphine derivatives, sulfenic acids, and a large variety of nucleophiles. Some of them have biological relevance (e.g., thiols and seleno compounds), but an important aspect of this broad reactivity is to provide perspectives for the development of efficient RSNOs detection methods and RSNO-based therapies—e.g., biotin labeling, phosphine compounds, and metal complexes (257–261). Noteworthy, low-molecular weight *S*-nitrosothiols derivatives such as *S*-nitroso-N-acetyl penicillamine (SNAP), GSNO and L-/D-CysNO are commonly used in biological experiments. For an in-depth description of RSNOs chemistry, very good articles, and reviews are available in the literature (254, 262, 263).

Considering their high biological relevance, mechanisms driving RSNO chemistry are matter of intensive investigations, and the ease of RSNOs dissociation through several pathways at room temperature, leading to the efficient release of nitric oxide, is an interesting property very often used in studies aiming at developing therapies against cancer and other diseases (264–271).

## Conclusions

The discovery of *S*-nitrosylation opened new venues in the context of the cellular signaling induced by post-translational modifications since NO relies on this modification to transmit its redox signaling. The mechanisms involved in its regulation and the effects caused by it are very complex and diverse, as described above. *S*-nitrosylation has been emerging also as a key mechanism in many diseases, such as cancer. As a result of extensive efforts by cellular and proteomics studies, we now know several enzymatic regulators of *S*-nitrosylation, as well as a myriad of protein targets that are modulated by this post-translational modification. However, structural studies that can help in understanding the mechanisms induced by this modification and its reactivity are still not as in-depth as the investigations carried out so far for other more conventional PTMs, such as phosphorylation. The complexity of this redox modification challenges experimental and computational structural and biophysical studies. Nevertheless, advances in computational biochemistry hold promise to both generate new mechanistic hypotheses that can be experimentally tested and rationalize at the molecular and atom level the experimental results collected so far. Several efforts are still required to experimentally solve new structures of *S*-nitrosylated proteins, using techniques such as X-ray crystallography and NMR, as well as to collect experimental data probes the conformational changes induced by this PTM on both structure and dynamics at local and distal sites of the proteins. Once more information will be available, we will be capable of overcoming the limitations of standard force fields to model the effects induced by *S*-nitrosylation on protein structure and dynamics, as well as to unveil the long-range allosteric effects triggered by this redox PTM. Similar studies can, for example, shed new light in the context of identifying and characterizing SNO sites that are buried in the native structure and that can become available for modification upon transient conformational changes of the protein. Moreover, in the context of reactivity, theoretical studies will need to account for more complete models that can account for the structural environment of the *S*-nitrosylated cysteine. Prediction algorithms for SNO-sites would also largely benefit from more available experimental and structural data, hopefully making the current predictions more accurate. The redox community should also dedicate more comprehensive, collaborative, and organized efforts toward the development of a common publicly available repository for the *S*-nitrosyloma with both sequence, structural and experimental information.

## Author contributions

ML contributed to the writing of chapter 3, MA to the writing of chapter 4, EB contributed to the writing of chapter 6 and 7 and MT to the writing of chapter 5. EP wrote the introduction, chapter 2, conclusions and contributed to the writing of all the other chapters. All the authors revised the final version of the manuscript and contributed with figures and tables.

### Conflict of interest statement

The authors declare that the research was conducted in the absence of any commercial or financial relationships that could be construed as a potential conflict of interest. The reviewer MS and handling Editor declared their shared affiliation.
